# Medico-Chirurgical Transactions

**Published:** 1817-12

**Authors:** 


(To be concluded in our next.)

---

*Medico-Chirurgical Transactions, Vol. VIII. Part I. Octavo, pp.* 315, *with Plates,* 1817.

Art. 1. *Report of the State of the wounded on board his Majesty's Ship Leander, in the Action before Algiers, &c.* By D. Quarrier, M. D. Surgeon of that Ship.

This is a very interesting document, and highly creditable to the talents, the zeal, and the humanity of Dr. Quarrier; whose skill and intrepidity we are happy to see rewarded by government, in the appointment which he now holds.*

As we have alluded to this report in our review of Mr. Hutchinson's very interesting pamphlet on Gun Shot Wounds, (a work which we cannot too strongly recommend) we shall be very explicit here.

"No language, says Dr. Quarrier, can pourtray the horrors of the Leander's cock-pit for a period of thirteen hours. Sixty five men were wounded and several killed by the first and second broadsides; two poor boys were most dreadfully burned by a red hot shot blowing up the cartridge which one of them was carefully guarding. The small space occupied for their accommodation was instantly crowded to excess: — without air; panting for breath; bathed in a most profuse perspiration; and unable to stand upright, these men were to be attended to: Water! water! was the incessant cry. Most fortunately an abundance had been provided, and the women supplied it liberally. Under these disadvantages and difficulties our operations were performed, and the poor patients were afterwards exposed to the double danger of being trampled upon by those who were rushing forward for relief." p. 2.

Our readers already know that Dr. Quarrier did not notice on this momentous occasion, that peculiar derangement of the sensorium which is said by some recent military surgeons to always attend wounds inflicted by large cannon shot. In our review of Mr. Hutchison's able dis-

* Surgeon to the Marine Artillery, at Fort Moncton.

cussion, (vol. iii. p. 492.) we glanced at the difference between naval and field practice in gun shot-wounds. The fact is, that on board a ship, the limb is often amputated before the *shock*, as it is called, has come on: for we believe it more frequently occurs half an hour after the accident, than at the very time.

"Six were amputated above the knee, some very high up, where we found the tourniquet useless; but there was no difficulty in restraining the hæmorrhage by the thumb, until the artery was secured." p. 4.

The following case must excite the feelings of a Stoic!

"Timothy Sullivan, seaman, had his left thigh most cruelly lacerated, the bone having been fractured up to its head, the nerves and blood vessels torn asunder. The crural artery was readily secured at the groin. His right arm was fractured, and he had a wound in the breast. Still he continued sensible, and made the most earnest supplications that I should operate on him. I declined it until Francis Coulthred, who had his right thigh shattered and carried away by a cannon ball, made such an appeal for him, that he could no longer be resisted. No! said this brave seaman, when he was going to be lifted, I am comparatively easy now, let me entreat you to render some assistance to that poor fellow who is suffering so much; he was a prisoner with me eight years. Amputation was consequently performed at the hip-joint, after the manner of M. Larrey; the vessels were readily secured, and he did not lose four ounces of blood; but, as I had anticipated, he expired almost immediately afterwards." p. 4.

Such traits of heroic disinterestedness in unsophisticated nature, like Oases in the deserts, refresh the languid eye of the philosopher, fatigued with the disgusting monotony of selfishness, egotism, and uncharitableness, which this world too generally presents.

Art. 2. *Cases of Hernia Cerebri, with Observations.* By Edward Stanley, Esq. Assistant Surgeon to St. Bartholomew's Hospital.

After adverting to the impropriety of the indiscriminate application of the terms "fungus cerebri" and "hernia cerebri" to all protruding tumours originating in the brain, whether they consist of mere coagulated blood, newly organized matter, or a part of the brain itself; and suggesting the restriction of the latter to those tumours which are really cerebral, Mr. Stanley relates the following cases.

Case 1. "A boy, aged 12, suffered an extensive fracture with depression, near the lambdoidal suture. He was trephined, the bone elevated, and the sequent inflammatory action subdued by local and general bleedings. Until the tenth day, every thing went on well. On removing the dressings at that period, a tumour was seen thrust up into the aperture in the cranium; and in three days, acquired the size of a small orange. A stratum of coagulum upon its irregular surface gave the tumour a dark colour, except at the centre, where the medullary substance of which it consisted, was evident. A visible vapour, and fœtid odour exhaled from it. Its pulsations were strong and regular. Pressure on it caused no pain. Febrile disorder, with anxiety of countenance, incoherent speech, and a degree of insensibility requiring strong excitement to obtain reply, accompanied, and increased with the tumour.

The plan of treatment adopted, was, to slice the tumour down on a level with the skull; to bring the edges of the scalp as near each other as possible by adhesive plaster; and to guard against future protrusion by gentle pressure. The boy did not appear to suffer pain during the operation. The bleeding from the cut surface of the brain, at first considerable, soon ceased.

The exterior of the excised part was coagulum; the remainder, brain. For two days after the operation, the boy was tranquil but on the third, complete insensibility, remarkable quickness the pulse, and strabismus, came on, and he died the following morning.

*Examination.* A coagulum, the size and thickness of a dollar, between the dura mater and bone, near the seat of the injury. In the vicinity of the ulceration, the dura mater was black, sloughy, and thickened. The structure of the surface, and for some distance beneath where the tumour had been removed, was softened, and broken down. Small sanguineous effusions between the membranes and in the substance of the brain. Tunica arachnoides thickened, and opaque. Vessels of the exterior and interior of the cerebrum remarkably empty. Ventricles filled with effusion. Fluid between the membranes; at the basis also. The fracture extended through the basis, to the foramen magnum.

Case 2. A depressed portion of the os frontis of a boy, aged 11 years, was raised by the application of the trephine and Hey's saw. The dura mater uninjured. Both arms fractured near the wrists, and the lower jaw broken at the symphysis. A soft substance resembling coagulum, of the size of a hazel nut, projected at the vacancy in the cranium, on the seventh day. Moderate pressure was applied to it, which restrained its rising, but caused it to expand laterally. The upper part of the tumour was pared off, and presented similar appearances with that of the last case. The boy's health remained undisturbed, but the protrusion increased notwithstanding the pressure applied. It was therefore cut down on a level with the bones, and found to consist entirely of medullary substance. Momentary pain was experienced as the knife passed through the tumour.

The pressure was increased by a graduated compress and bandage, and borne without complaint for three days; but still the tumour had risen in a trifling degree. The disposition to protrude however, soon ceased, and the projecting brain acquired a yellow colour, split into fissures, became almost fluid, and gradually wasted away. Granulations, arising from the brain beneath, filled up the vacancy; so that the space formerly occupied by the protrusion was soon cicatrized, and the boy well."

CASE 3. "By the application of the trephine, and removal of bone, an aperture, of three inches by two, was made in the cranium of a boy, aged 13, who had his os frontis fractured by a kick from a horse. Inflammatory action supervened; but on the fourth day from the operation, he was free from complaint. On inspecting the wound at this time, the dura mater (which in this case also was uninjured by the accident) was seen slightly raised in the aperture. In two days more, it had risen above the level of the skull; was tense, black, and mortified at two points, which soon gave way, and small protrusions projected at each opening. On the eighth day, the protruding mass equalled the size of an egg; the intellects and health remaining unimpaired. The treatment of this case was similar to that of the preceding. Considerable pain was felt during the operation. Excision and pressure were followed by sloughing and granulation; but when the wound became one continuous granulating surface, symptoms of compressed brain rendered it necessary to apply the bandage less firmly; and in consequence, by the next day, the brain had again risen above the level of the skull. Insensibility; nervous disorder; loss of voluntary motion; and convulsive twitches of the left arm, precluded the renewal of the pressure. During the last three days of the boy's life, there was a constant oozing of serum from the centre of the protrusion. He died on the twenty-seventh day after the accident."

*Examination.* "The tumour evidently consisting of medullary substance. *Externally*, shrunk, black, and sloughy. *Internally*, soft, with sanguineous effusion. Pus spread over the arachnoid membrane, particularly on the left side, and lodged on each side the falx. For some distance around the tumour, the dura mater and brain were separated so as to leave a considerable space.* The greater part of the medulla of the right hemisphere was soft, pulpy, and rotten. The substance of the brain surrounding this disorganization, retained its healthy firmness, but was of a greyish blue colour. Three ounces of fluid were collected from the ventricles, and between the membranes. The extent of the fracture was from the frontal bone to the basis of the skull on the right side."

The author next enquires into the cause producing pro-

* We think it probable, that this cavity, during life, was occupied by the matter which, post mortem, was found spread on the arachnoid membrane—the shrinking of the parts allowing it an exit. EDIT.

trusion of the brain; observing, that it is not the mere want of pressure from loss of bone to which it is to be attributed, but to an increase of the cranial contents by distention of vessels, or effusion. The following observations were made in Case 2.

"When the boy was lying quietly in bed, the motions of the protruded brain accorded regularly with the pulsations of the arteries. When he rose, the tumour instantly sunk to a certain degree, probably from the blood being then returned more freely from the head than when in the horizontal position. When he was desired to hold his breath, the nostrils being at the same time closed, no alteration in the tumour was produced. In inspiration, preceding the act of coughing, the brain sunk; but in the instant of the forcible expiration, it was again driven upwards with great force."

With a view of preventing the occurrence of protrusion in cases of fracture, pressure, by tight bandage or other contrivance, equivalent to that of the skull itself, should, from the commencement, supply the deficiency of cranium; whilst great attention is necessary to prevent increase in the volume of the brain, distention of its vessels, or effusion.

A case of hernia cerebri, related in Van Swieten's commentaries, in which the protruded brain was removed by ligature:—another in the Mem. Acad. François, in which the patient himself tore it off, in a state of intoxication: another in the Medical Commentaries, in which it spontaneously dropt off;—a fourth, related by Mr. Pring in the Edinburgh Journal, in which it was removed, and pressure applied:—and a fifth, by Mr. Taylor, Surgeon to the forces, in which pressure only was made use of;—all terminated favourably.

An interesting case, communicated by the direction of Mr. Pearson, the particulars of which we shall briefly detail, closes the article before us.

"A man, aged 20, came into the Lock Hospital with nodes on the os brachii and tibiæ, a tumour on the forehead, which appeared to contain fluid, and an ulcer in each tonsil. The tumour was repeatedly punctured, and a small quantity of fluid, somewhat thicker than serum, discharged each time; but without benefit. It was, therefore, divided by incision, and the frontal bone found carious. The wound went on well, discharging considerably for about three weeks, when symptoms of compression suddenly occurred, but subsided entirely in twelve hours, by venæsection, purging, and fomentations. Another similar attack yielded to similar treatment.

"The carious bone was now easily removed by the forceps. The exposed surface was "a diseased, dark coloured mass, projecting beyond the level of the opening." This gradually increased; the dark coloured sloughs separating it, assumed the appearance of "flesh somewhat vascular," and had a pulsatory motion. No advantage was derived from pressure. The patient now accidentally struck his head with such violence, as to break a considerable portion of the tumour, which hung pendulous, and was removed by the scissors without hæmorrhage, but followed by a discharge of a table-spoon full of fluid, similar to that found in the lateral ventricles. Still the tumour increased. A strong ligature at its base, tightened every day, retarded not its advance. The appetite, sleep, and locomotion, remained all this time nearly the same as before the accident. By a similar accident to that which has been already mentioned, another large portion of the tumour, in "a corrupted state," and having little the appearance of brain, was detached. The day after this, "the man's strength visibly decreased"; stupor, insensibility, with dilated pupils, appeared suddenly after breakfast the following morning, and he died in the afternoon.

The dimensions of the tumour before the ligature was applied, were, — longest diameter, six inches and a half; — shortest diameter, five inches and a half;—elevation above the aperture, two inches.

*Examination.* Dura mater adhering to the edges of the aperture. The "morbid mass" firmest at its surface, evidently continuous with the right hemisphere of the cerebrum. Ventricles contained some ounces of bloody fluid. An abscess, containing two or three ounces of pus, in the anterior lobe of the right hemisphere.

Art. 3. *History of a Case of Rupture of the Brain and its Membranes from Hydrocephalus Internus.* By John Baron, M. D. Physician to the Infirmary at Gloucester.

It occurred in a girl, about 4 months old. The circumference of the head when first measured, was 28, and in a week 29 inches; the bones being considerably separated. No increase of circumference took place; but a swelling, the size of a goose's egg, appeared over the posterior fontanelle. This disappeared in consequence of a continual discharge of water from the urinary passages; and the formerly distended skin fell in wrinkles over the forehead and eyes.

An increased discharge from the bladder continued two months. On its subsiding, the head rapidly enlarged; the swelling extending itself over the whole head and part of the face. An oozing of an aqueous fluid, tinged with blood, from the mouth and nostrils, which continued till the child's death, again diminished the head, and prevented re-accumulation.

A temporary suspension of the oozing from the nostrils was followed by an increased renal secretion. On holding the head forwards, the fluid freely discharged ex naso.

The child continued sensible, ate well, and had natural alvine evacuations to the last; but increased not in size from the time she was first seen.

*Examination.* Circumference of the head, 20 inches. A little on the right of the falx there was a circular aperture in the dura mater, forming a communication between what was the external tumour, and a cavity formed in the interior of the brain by the expansion of its hemispheres, containing three or four pints of fluid.

"The expansion of the brain was so great, that round the margin of the opening in the dura mater, it did not equal the thickness of a shilling; and, under the opening, it had entirely disappeared; proving, that it had given way when the dura mater yielded, and allowed the fluid from the internal cavity to escape with the outward swelling."

Art. 4. *Observations on the Morbid Structure of Bones, and Attempt at an Arrangement of their Diseases.*— By J. Howship, Esq. M. R. C. S. and corresponding Member of the Societé Medicale de Emulation, in Paris.

Mr. Howship pursues this interesting subject, to which he has already devoted much attention, with his usual talent for observation, and industrious inquiry. The nature of the paper before us, and our prescribed limits, do not admit of its analysis; we therefore, particularly recommend the perusal of these observations to our readers, assuring them, they will find in this, as in all the communications from Mr. H. much worthy their notice.

Art. 5. *An Inquiry into the Origin and Nature of the Yellow Fever, as it has lately appeared in the West Indies, &c. &c.* By William Fergusson, M.D. &c. &c.

The beginning of this paper is occupied with the history of the Regalia transport, which has already been detailed in this Journal by Mr. Mortimer, *vol. iii.* Dr. F. traces the fever to the same source as Mr. Mortimer — green wood, and foul ballast. Our author next takes up the subject of yellow fever, and, in opposition to Drs. Chisholm and Pym, shews it to be of endemic origin, noncontagious, and capable of affecting the system twice or oftener. In all his reasonings on these subjects, we see good sense, discrimination, and clear judgment; but we can see very little in addition to what many good writers have already said on these topics, and particularly Dr. Dickson, whose experience and talent for investigation give high value to his conclusions.*

There are some points, however, on which our author differs from several of the most respectable of the anti-contagionists, namely, the identity, or non-identity of concentrated yellow fever with bilious remittent fever. Dr. M'Arthur, whose authority is strong, is well known to be of opinion, that the fever in question, is different from the remittent; and we suspect that Dr. Dickson and some other able writers, in applying the epithet "*continued,*" to this fever, give grounds for disjoining it from the *remittent* family.* On the other hand, our author reasons thus —

"To the argument, that the highest degree of concentrated remittent or yellow fever, should neither remit nor break off into ague, it seems sufficient to reply, that for any disease to observe regular laws, it is necessary that the vital organs principally affected should continue in a certain degree of integrity; that their functions should only be perverted and disturbed to a given point; that they should still be discernible as functions, and not be utterly overwhelmed and extinguished by the violent cerebral action and speedy gangrene of the stomach that takes place in aggravated yellow fever. As the ulcer of a specific poison that would run a regulated course according to acknowledged laws, if it be driven to a high inflammation or sphacelus, no longer belongs to the original stock, and is emancipated from those laws; so the violent actions of the above fever impair and destroy the animal functions by which its crises and remissions are regulated, or speedily engender a new disease; as new as the conversion of an ordinary venereal chancre into a phagedenic slough, through the application of a potential cautery.

"I feel that this almost inscrutable subject is at present greatly beyond my depth. I wish only to shew, that the difficulties of reconciling the phænomena of remittent with what has been called yellow fever, are not so great as what has been represented, nor greater than what exist in many other diseases, respecting the identity and origin of which the same difficulties have not been started. The difference between the different degrees of the intertropical fever, that are characterized by irritability of stomach, and ter-

* Vide this gentleman's papers in the Edinburgh Medical and Surgical Journal.

* Edinburgh Medical and Surgical Journal for January, 1817, page 49.

minate occasionally in discolourations of the skin, or what have been called, remittent, bilious remittent, and Bulam, are surely not greater than what we see daily in another disease at home, which, however, is propagated by different laws. There scarlatina will attack one patient in a form so mild, that but for the subsequent desquamation of the cuticle, it would be difficult to detect its existence. Another will have vivid eruptions and high phlogistic symptoms, while a third exhibits a low putrid malignant plague, with gangrenous sore throat, and scarcely any eruptions at all. No one thinks of doubting the identity of the disease in these three conditions, because they have all one common property, the capability of communicating the infection to others; but this last is not an essential quality of many other acute distempers, nor is it more characteristic than that of the Bulam fever being limited almost exclusively to the unseasoned and the stranger. It is surely more probable that the endemic causes of disease should operate upon their unseasoned bodies with peculiar severity, so as to produce something more than an ordinary remittent fever, and that they should remain altogether exempted from their operation, and even while the Creoles and the seasoned are suffering their limited share of endemic ills, be amenable solely to a peculiar contagion, reserved altogether for their use, or imported with incomprehensible punctuality for the occasion of armaments newly arrived, sultry calms and long continued droughts, by which the exhalations from the earth are known to be sublimed into peculiar activity." 137.

We acknowledge that there is much good reasoning in the foregoing passage. Dr. Fergusson, contrary to the statement of Dr. Bancroft, believes that contagious or typhoid fever may exist between the tropics.

"*I have no doubt but that a typhoid infection may exist here, the same as elsewhere, which, however, is certainly dissipated as soon as ventilation and purity are restored. This was exemplified in the* Childers *brig, that lately arrived at Barbadoes in so distressing a state from Trinidad. The fevers on board of her, from crowding below decks when at sea, ceased to be yellow ones, and became as truly typhoid as any I ever saw. That the ship was impregnated with a typhoid contagion, capable of infecting others within its sphere, I have little doubt; and am sure, from what I have seen, that crowded hospitals on shore may be brought for a time to the same dangerous condition from undue accumulation of sick, even in these (West India) climates.*" 153.

We have said, that we fully coincide with Dr. Fergusson in all his remarks on the locale, &c. of yellow fever, because they are consonant with our own experience; and what is still more, because they run parallel with what some of our ablest and keenest observers have already stated.*

* See particularly Dr. Dickson's last paper in the 13th vol. Edinburgh Journal.

But, after travelling agreeably together till the goal of our journey was in view, we are reluctantly compelled to turn short off, and take separate roads. After disproving infection in the Regalia transport, and insisting that—"there was nothing in her, nor about her, that could either generate or permit the retention, if introduced, of the matter of typhus fever," p. 116, we were not a little surprized to peruse the extraordinary passage quoted above, in respect to the Childers brig. What! does a British man of war hatch infection, which could not be generated, or even live, under circumstances so much more favourable to it, namely, in *a slave ship?* This is really almost a libel on Naval discipline. Allowing the sloop to be the filthiest in the service, she could be only *relatively* so; and, far from equalling, in this respect, in crowding, and in want of ventilation, those in *other services.* Surely, then, on all these points, she could come nothing near the state of congregated animal effluvia in the hot-bed of a slave ship! Again, the Childers had been six months in the West Indies; *healthy;* consequently she could not be supposed to have brought the infection with her. But were we to grant its generation in that climate, is *that* likely to happen in a British man of war, between Trinidad and Barbadoes, *which,* a passage from Africa to the West Indies, in a sickly crowded transport, was inadequate to effect?

That the fevers in the Childers put on a mere *typhoid* appearance, owing to crowding, and probably to *treatment* at sea, we are ready to admit; but that they were therefore *typhous* and infectious, we cannot grant. If we are to believe in this agency in the one case, and disbelieve it in the other, where circumstances were so much more favourable to it, we must say, like one of the ancient fathers of the church, "credo quia absurdum"—now dicam—"quia impossibile." We consider the Childers as in a similar state with the Dart sloop of war in 1807, as described by Dr. Dickson in the *January Number of the Edinburgh Journal, page 36.* The source of the fever was strictly *endemic*—it was as Mr. Birnie, the assistant surgeon of the vessel, says, in the hold and lower parts of the ship; and by a six month's preparation, by accumulation of predisposition or susceptibility in the ship's crew, and an epidemic state of the season, its morbific power had attained a maximum, superior, in general and destructive influence, (from the above combination of circumstances) to any individual, or, we might say, any infectious agency whatever. We believe that even the supporters of the Bulam fever," a highly contagious, monstrous, new, and destructive disease," would hardly contend that the Nova Pestis would spare but *three* out of *ninety* men. In fine, every thing shews the disease to have been *in*, but not transferrable *from* the ship, viz. men taken ill [and fresh ones, even black Creoles from the shore] every day until unloaded and cleared out; officers and others successively attacked, after being some time on board. No new case of fever after purification; no instance of its communication to those on shore.

We are indeed utterly astonished how a man of Dr. Fergusson's penetration could fail to see, at a single glance, that his conclusion, relative to the Childers, has furnished the contagionists with the best materials for weakening and discrediting all his preceding statements! If such agency undoubtedly existed in the Childers, with how much stronger reason might it be inferred in the Regalia? We beg of Dr. Fergusson to re-consider these things, and not to allow the "*tenax propositi*," as is too often the case in medical science, to shed its *Procrustes* influence over an investigation, involving such important concerns in the welfare of the human race!

